# A fatal retroperitoneal bleeding from iliolumbar artery following open reduction and internal fixation of an unstable pelvic ring injury: A case report

**DOI:** 10.1097/MD.0000000000032798

**Published:** 2023-02-17

**Authors:** Suk-Kyoon Song, Jinkyu Park, Sungho Lee

**Affiliations:** a Department of Orthopaedic Surgery, Daegu Catholic University Hospital, Daegu, Republic of Korea; b Department of Orthopedic and Traumatic Surgery, Cheju Halla General Hospital, Jeju-si, Republic of Korea.

**Keywords:** abdominal compartment syndrome, iliolumbar artery, pelvic ring injury, retroperitoneal hemorrhage

## Abstract

Retroperitoneal bleedings are the result of injury to parenchymal tissue or vascular structures within retroperitoneal cavity. This may arise spontaneously, as a result of trauma or iatrogenically. Patients usually do not manifest clinically apparent signs and symptoms until a substantial amount of blood loss has occurred. Therefore, it is associated with high morbidity and mortality. Herein we present and discuss a case of fatal iatrogenic retroperitoneal bleeding following open reduction and internal fixation of an unstable pelvic ring injury.

**Patient concerns:**

A 66-year-old female patient presented to the Level I regional trauma center with severe pelvic pain after a pedestrian collision by a car.

**Diagnoses:**

In initial radiography and computed tomography, she was diagnosed with unstable pelvic ring injury.

**Interventions:**

Definitive surgery for open reduction and internal fixation through the anterior approach to the sacroiliac joint and anterior intrapelvic approach was performed on the 8th day after the injury.

**Outcomes:**

Patient died 3 days after the surgery due to a massive retroperitoneal bleeding from iliolumbar artery.

**Lessons:**

Insidious retroperitoneal bleeding from the small vessel may lead to fatal massive retroperitoneal hematoma. Therefore, active retroperitoneal bleeding should be suspected in cases of unexplained unstable hemodynamic status following orthopedic pelvic and acetabular surgery.

## 1. Introduction

Retroperitoneal bleeding is a hemorrhage into the retroperitoneal space, the space located behind the posterior reflection of the parietal peritoneum.^[1]^ Clinically retroperitoneal bleeding can present with diffuse abdominal, back or lower quadrant abdominal pain, abdominal distension and palpation of a flank mass.^[2,3]^ However, these clinical findings are nonspecific for the diagnosis and further contribute to the difficulty in diagnosis. Patients usually do not manifest clinically apparent signs and symptoms until a substantial amount of blood loss has occurred.^[1]^ Therefore, it is associated with high morbidity and mortality. The management of retroperitoneal bleeding depends on the cause and size of bleed, hemodynamic status and stability of the patient, and the presence of the active bleeding.^[3,4]^ Treatment modalities include observation, interventional radiology coiling/embolization, and operative management for unstable patients.^[5,6]^ Retroperitoneal bleeding of significant volume can be concealed in the potential space and the result in hypovolemic shock, necessitating transfusion with urgent angiographic or surgical treatment.^[1]^ Retroperitoneal bleeding can occur spontaneously, or be secondary to trauma. Retroperitoneal bleeding can also be iatrogenic, as a complication from surgeries or transfemoral catheterization procedures.^[1,2]^ In particular, pelvic fracture surgery is associated with various retroperitoneal vascular injuries and they may lead to fatal outcomes. Herein, we report a case of fatal retroperitoneal bleeding from the iliolumbar artery (ILA) following open reduction and internal fixation of an unstable pelvic ring injury through the anterior approach to the sacroiliac (SI) joint.

This trial was conducted after approval of the Cheju Halla General Hospital Institutional Review Board (2022-L11-01). The requirement for informed consent was waived, as all data were de-identified to protect patient’s right to privacy.

## 2. Case

### 2.1. History and presentation

**Figure 1.**
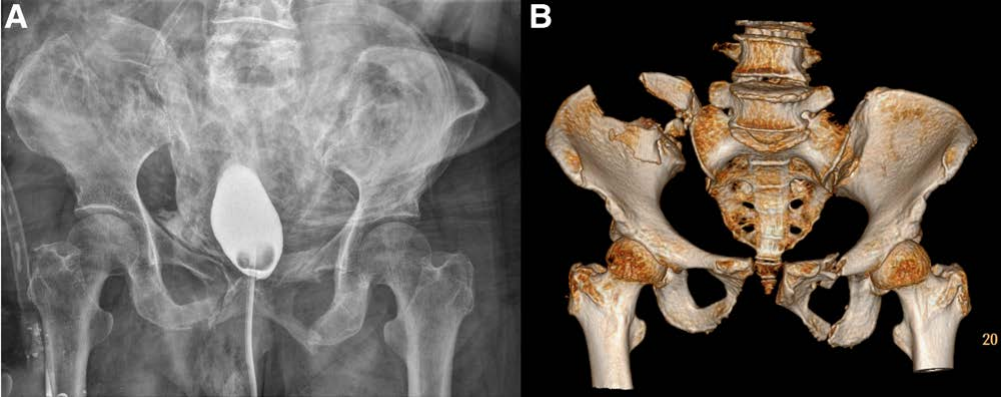
Initial radiographs. (A) Pelvis AP x-ray and (B) 3D reconstruction of computed tomography, revealing unstable pelvic ring injury. AP = anteroposterior.

**Figure 2.**
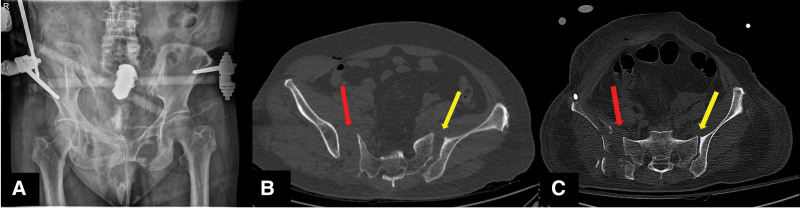
Damage control external fixation. (A) Pelvis AP x-ray demonstrating volume reduction of the pelvic cavity (B) axial view of computed tomography demonstrating anatomic reduction of the left SI joint (yellow arrow), still widening of the right SI joint (red arrow) compared with (C) preoperative image. AP = anteroposterior, SI = sacroiliac.

A 66-year-old female presented to the Level I regional trauma center with complaining of chest wall pain and severe pelvic pain following a pedestrian collision by a car in a squatting position. Medical comorbidities included well-controlled diabetes mellitus and hypertension. Her regular medications did not include blood thinners such as anti-platelet agents and anticoagulants. The patient had no history of a bleeding diathesis. Pelvic radiography showed an unstable pelvic ring injury consisting of right SI joint fracture/dislocation with an iliac crescent fragment, parasymphyseal pubic ramus fractures, opening of the anterior SI joint, and diastasis of the symphysis pubis (Fig. 1). Other findings were included multiple rib fractures without flail chest. No evidence of visceral organ injury was found. We planned anterior external fixation for anterior pelvic ring stability and iliosacral (IS) screw insertion for posterior pelvic ring stability for damage-control surgery. However, the iliac injury extended through which IS screw would be placed. Therefore, external fixation of the anterior ring alone, without posterior fixation, was performed (Fig. 2). The immediate postoperative hemodynamic status and vital signs were stable, except for slightly decreased hemoglobin level. The patient was transferred to the general ward following close observation in the trauma intensive care unit for 2 days. The patient was alert, hemodynamic status, and vital signs were stable. We planned definitive surgery for open reduction and internal fixation on the 8th day after the injury.

### 2.2. Surgical planning

1. IS screw fixation of the left SI joint prior to the removal of the external fixator.
2. Open reduction and internal fixation with plates for the right SI joint fracture/dislocation through the anterior approach to the SI joint.
3. Open reduction and fixation of the anterior pelvic ring through the anterior intrapelvic approach using a Pfannenstiel incision.

### 2.3. Operation

**Figure 3.**
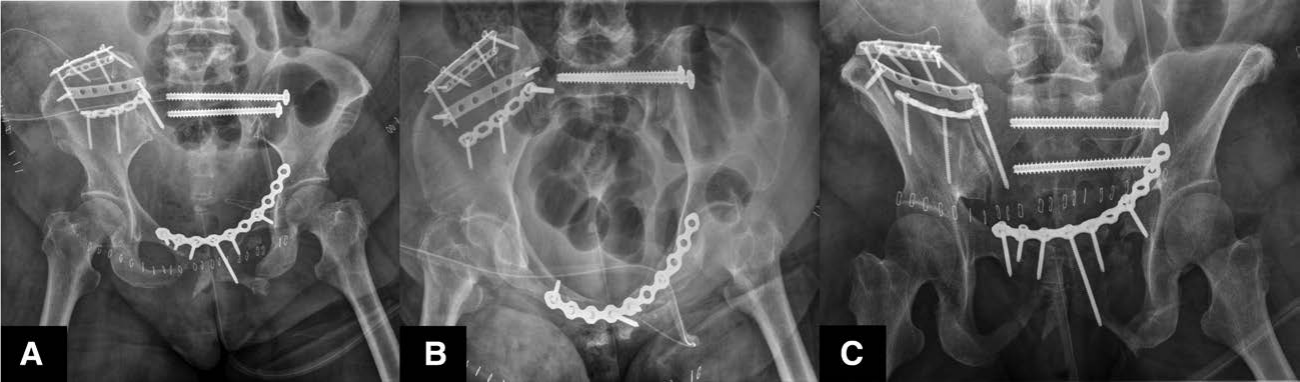
Definitive fixation state. (A) Pelvis AP, (B) inlet, (C) outlet views revealing restoration of the pelvic ring and anatomic reduction of both SI joint. AP = anteroposterior, SI = sacroiliac.

On the day of surgery, the patient’s hemodynamic status and vital signs were stable. Surgery was performed under general anesthesia. Left IS screw fixation was performed prior to the removal of the external fixator. For the anterior approach to the SI joint, an incision was made along the iliac crest, extending posteriorly, almost to the table. Incision was made through the muscular interval between the gluteus muscle and external oblique muscle with unipolar electrocautery. The external oblique muscle was subperiosteally elevated from the iliac crest using a Cobb’s elevator, the iliacus muscle was elevated using the same subperiosteal layer. When elevating the iliacus muscle, bleeding from nutrient vessels was stopped by bone wax or electrocautery. Careful blunt dissection was continued to the SI joint. A disrupted SI joint was identified and placed a Hohmann retractor was placed in the superior portion of the SI joint. The iliacus muscle and L5 nerve root were safely reflected using a malleable retractor without excessive tension. No visible active bleeding was observed. The SI joint was distracted by using a laminar spreader. The joint was inspected and small fragments and interposed soft tissues were evacuated. Reduction of the SI joint was performed combination of using longitudinal traction, joystick maneuver by a large bone holding clamp for the iliac wing, pointed reduction forceps on the sacrum, and the ilium. Provisional fixation with percutaneous 1.6 mm K-wire was performed to maintain of reduction. The 2 plates were used to fix the right SI joint. Two other plates were used to fix the iliac crescent fragments. No retractor was placed in the greater sciatic notch to avoid major neurovascular injury. No active bleeding or oozing was observed at any stage of the procedure. The aponeurosis of the external oblique muscle was repaired and the surgical wound was closed in layers with the insertion of a drain tube. The anterior intrapelvic approach was performed using a Pfannenstiel incision with a standard deep dissection. Reduction and fixation with a pelvic brim plate were performed, without any remarkable events (Fig. 3).

### 2.4. Postoperative course

**Figure 4.**
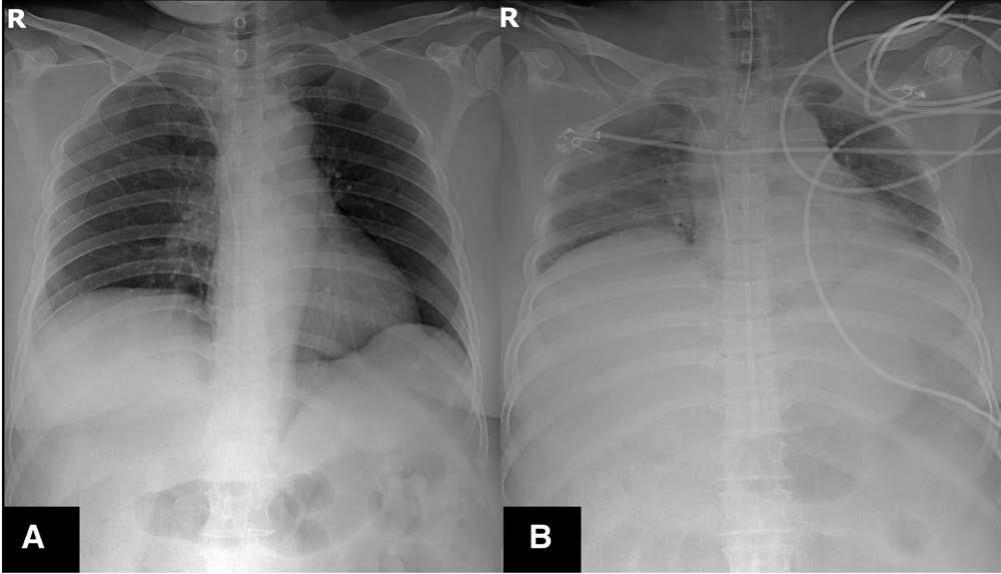
(A) Chest radiography revealing definite abdominal distension with lung volume reduction compared with (B) preoperative image.

**Figure 5.**
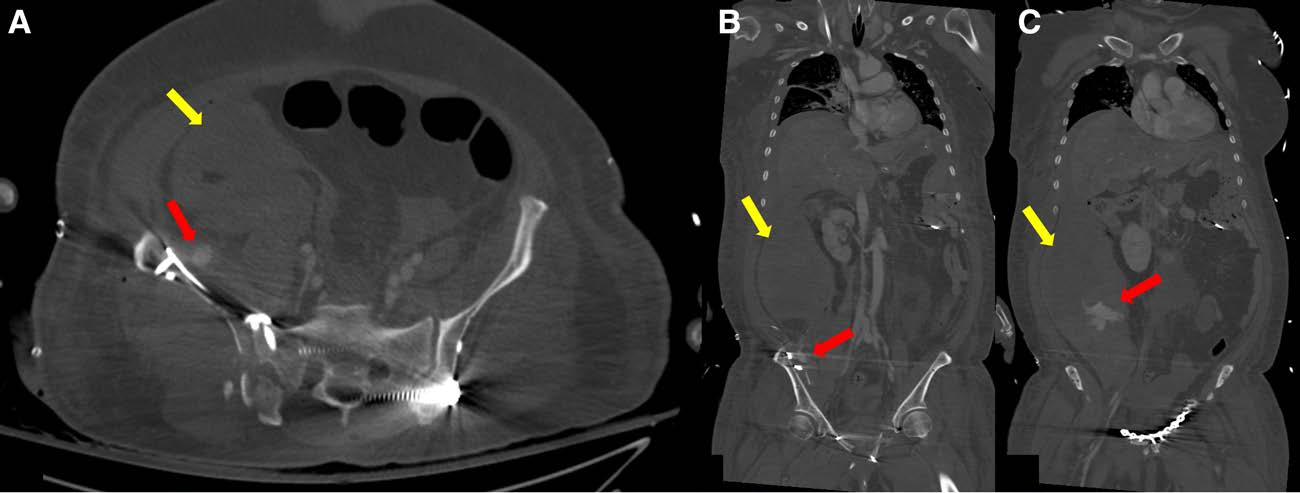
(A) Coronal and (B, C) axial views of computed tomography demonstrating a massive hematoma (yellow arrows) with extravasation of contrast media (red arrows).

**Figure 6.**
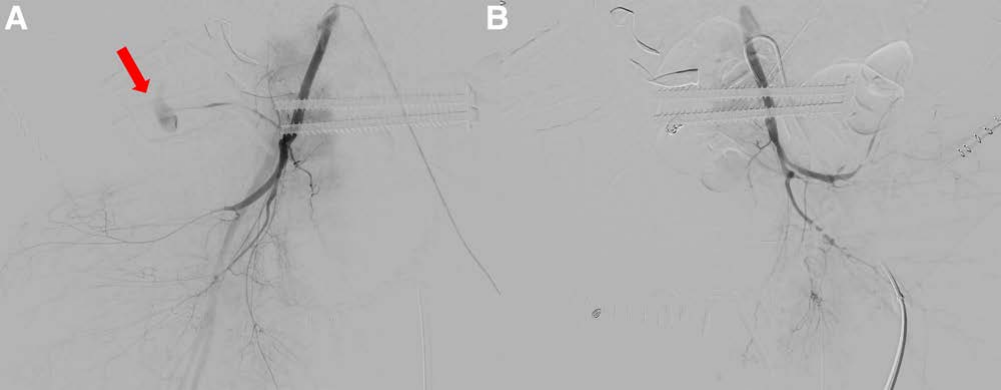
(A) Angiography revealing active extravasation from the right iliolumbar artery (arrows) compared with (B) contralateral side.

The total operation took 7 hours. Immediate post-operative vital signs revealed intermittent tachycardia and hypotension. Eight hours after the surgery, the patient had been developed abdominal distension with respiratory distress. The patient’s vital signs had deteriorated. Urgent chest radiography revealed abdominal distension with reduced lung volume (Fig. 4). Urgent computed tomography (CT) revealed a massive retroperitoneal hematoma with active extravasation of blood around the iliac fossa (Fig. 5). Urgent angiography confirmed extravasation of blood originating from the ILA (Fig. 6). Coiling of the internal iliac artery (IIA) was performed bilaterally to prevent further bleeding. Surgical decompression laparotomy was performed to evacuate the hematoma. An explorative anterior approach to the SI joint was performed simultaneously. No visible active bleeding was observed beneath the iliacus muscle. We found that the hematoma was connected to the retroperitoneal space of the pelvic cavity. Despite every effort to resuscitate, the patient already developed multiple organ failure. Three days after the definitive fixation surgery, the patient expired.

## 3. Discussion

Retroperitoneal bleeding is defined as bleeding into the retroperitoneal space that space lies directly posterior to the peritoneal cavity. Although the mainstay of diagnosis for retroperitoneal bleeding is a contrast-enhanced CT-scan,^[1]^ the diagnosis requires a high degree of clinical suspicion. The physical examination is commonly non-diagnostic at best, and they are not readily detectable on plain radiography or ultrasound.^[1]^ Therefore, retroperitoneal bleeding is a cause of significant morbidity and mortality. This is divided into traumatic and non-traumatic. The traumatic can be further subdivided into penetrating and blunt. The non-traumatic category can be further subdivided into spontaneous and iatrogenic.^[5]^ The etiology of iatrogenic retroperitoneal hematoma can be related to complication of surgeries or percutaneous transfemoral catheterization procedures.^[1,2,7]^ As see in this case, major vascular injury as a complication from pelvic fracture surgery may lead to fatal outcome. Most bleeding during pelvic fracture surgeries are from the branches of the IIA and the fractured bone.^[8]^ The ILA is one of a branch of the posterior division of the IIA. However, the origin variability of ILA has been reported. Al Talalwah et al^[9]^ reported the incidence of 3 different sources of origin of ILA which are posterior division of the IIA, trunk of the IIA, and common iliac artery. The ILA is divided into the lumbar branch and the iliac branch. The iliac branch passes underneath the iliacus muscle in the iliac fossa to supply to the iliac crest or around the SI joint. Therefore, the ILA and the branch supplying ilium are vulnerable to injury during anterior approaches to the SI joint for internal fixation.^[10]^ It should great care not to injure the vessel and perform meticulous hemostasis in around the SI joint and iliac fossa. In the present case, despite the absence of intraoperative active bleeding during the operation and no postoperative active drainage from the placed drain tube, insidious onset of retroperitoneal bleeding, massive retroperitoneal hematoma followed by abdominal compartment syndrome, respiratory distress was developed sequentially. This vicious progression contributed to the rapid deterioration of the patient’s condition. It is believed reduction and stabilization of the pelvic ring results in hemostasis of retroperitoneal bleeding by tamponade like effect. However, self-tamponade of retroperitoneal bleeding can fail despite internal fixation. Severe damage of the constraining ligaments of the pelvic ring and pelvic floor in high energy blunt trauma, such as an unstable pelvic ring injury, hampers effective retroperitoneal self-tamponade. Therefore, retroperitoneal bleeding can drain through the ruptured pelvic floor into the abdomen or chest.^[11–13]^ As seen with this case, insidious retroperitoneal bleeding from the small vessel may lead to fatal massive retroperitoneal hematoma.

In conclusion, massive retroperitoneal bleeding should be suspected in cases of unexplained unstable hemodynamic status following orthopedic pelvic and acetabular surgery. The diagnostic gold standard for retroperitoneal hemorrhage is CT of the abdomen and pelvis. Prompt diagnosis and management are critical and urgent surgical decompression is required to prevent the abdominal compartment syndrome and organ failure.

## Author contributions

**Conceptualization:** Sungho Lee.

**Formal analysis:** Jinkyu Park, Sungho Lee.

**Supervision:** Sungho Lee.

**Writing – original draft:** Suk-Kyoon Song, Sungho Lee.

**Writing – review & editing:** Suk-Kyoon Song, Jinkyu Park, Sungho Lee.
